# Impact of *TNF-α* (rs1800629) and *IL-6* (rs1800795) Polymorphisms on Cognitive Impairment in Asian Breast Cancer Patients

**DOI:** 10.1371/journal.pone.0164204

**Published:** 2016-10-04

**Authors:** Jung-woo Chae, Terence Ng, Hui Ling Yeo, Maung Shwe, Yan Xiang Gan, Han Kiat Ho, Alexandre Chan

**Affiliations:** 1 Department of Pharmacy, National University of Singapore, Singapore; 2 Department of Pharmacy, National Cancer Centre Singapore, Singapore; 3 Duke-NUS Graduate Medical School Singapore, Singapore; University of San Francisco, UNITED STATES

## Abstract

**Objective:**

Expression of pro-inflammatory cytokines is influenced by single nucleotide polymorphisms (SNPs) in the promoter regions of the pro-inflammatory cytokine genes, and cytokines are associated with the occurrence of post-chemotherapy cognitive impairment. Hence, the aim of this study was to evaluate the associations between two common pro-inflammatory cytokine gene polymorphisms namely, *IL6-174* (rs1800795 G>C) and *TNF-308* (rs1800629 G>A), and chemotherapy-associated cognitive impairment (CACI) among Asian early-stage breast cancer patients. In addition, the differential effect of these SNPs on plasma IL-6 and TNF-α levels, and the associations of plasma IL-6 and TNF-α levels with CACI were also assessed.

**Methods:**

Asian early-stage breast cancer patients (Stage I to III) receiving chemotherapy were prospectively recruited from two cancer centers in Singapore. Patients' cognitive function was longitudinally assessed using the validated FACT-Cog (ver. 3) and an objective computerized battery, Headminder™ at three-time points. Plasma IL-6 and TNF-α levels were analyzed using the multiplex immunoassay, and genotyping was performed using Sanger sequencing. Regression analyses and generalized estimating equation were utilized for statistical analysis.

**Results:**

A total of 125 patients were included (mean age: 50.3; Chinese: 80.8%; post-menopausal: 48.0%; 68.0% received anthracycline-based chemotherapy). 36.8% patients experienced self-perceived cognitive impairment, detected in memory (32.8%) and attention (34.2%) domains. Patients with higher levels of anxiety (p<0.001) and insomnia (p = 0.003) also reported more self-perceived cognitive impairment. Higher plasma concentrations of IL-6 were associated with greater severity of self-perceived cognitive impairment (p = 0.001). Polymorphisms of cytokine genes were not associated with expression of plasma cytokines.

**Conclusion:**

Present findings further contribute to the growing evidence that supports the role of the pro-inflammatory cytokine IL-6 in the occurrence of cognitive impairment post-chemotherapy. However, genetic polymorphism of these cytokines did not play a major role to the cytokine fluctuations as well as cognitive impairment in this cohort. With an increasing evidence to support the cytokine hypothesis, future studies should investigate the role of anti-inflammatory agents in mitigating the cognitive impairment associated with chemotherapy.

## Introduction

On the physiological level, inflammatory cytokines play important roles in the central nervous system (CNS) to modulate neuronal and glial cell functioning and neuronal repair [1–3]. However, the use of anticancer treatment coupled with patients’ psychological distress could trigger the inflammation cascade, which could lead to cytokine dysregulation. In patients with cancer, such inflammation cascade is speculated to increase one’s risk for subtle changes in cognition. This phenomenon is also known as cancer-associated cognitive impairment (CACI) or ‘chemobrain’ in the literature.

Evidence suggests that expression of pro-inflammatory cytokines is influenced by single nucleotide polymorphisms (SNPs) in the promoter regions of the pro-inflammatory cytokine genes. For example, the *TNF-308* G>A (rs1800629) polymorphism has been associated with high transcriptional activity, which increases the secretion levels of TNF-α, whereas the *IL6-174* G>C (rs1800795) polymorphism has been reported to influence the expression of IL-6, with carriers of the G allele being associated with higher plasma concentrations of IL-6 [4–6]. Few studies have been performed to evaluate the association between these SNPs and cognitive impairment in cancer patients. One study reported that carriers of the G allele of *IL6-174* G>C (rs1800795) were associated with poorer attention [7]. Another study suggested that carriers of high-expression alleles of *IL6-174* and *TNF-308* were associated with poorer memory in breast cancer patients [8]. Taken together, current findings suggest that host genetic factors could contribute to differential expression of the pro-inflammatory cytokines, which in turn translate to varying degrees of susceptibility to CACI among patients.

**Fig 1:**
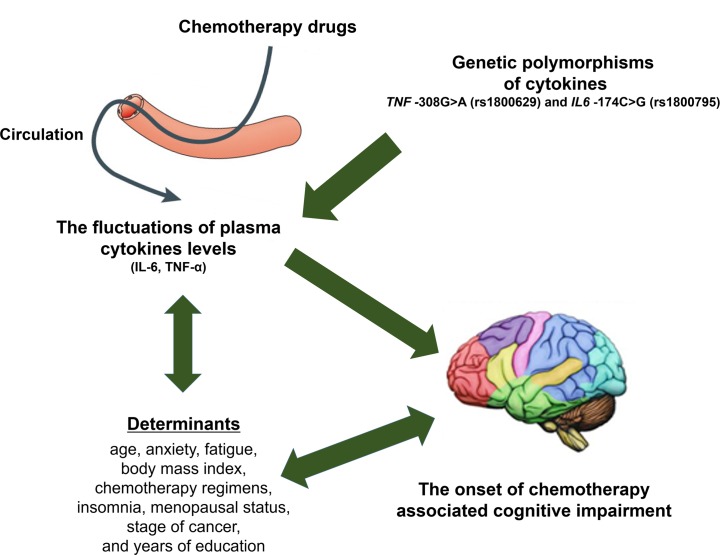
A conceptual framework including determinants that might affect cognitive impairment in chemotherapy-treated breast cancer patients. This study was performed to evaluate the downstream effects of *TNF-308* and *IL6-174* polymorphisms on the circulating TNF-α and IL-6 levels, and the occurrence of chemotherapy-associated cognitive impairment.

Singapore is a multiracial country with a majority population of Chinese, Malay and Indian, with English and Chinese being the most commonly spoken languages. As genetic polymorphisms are known to be demographic specific, it would be of great interest to determine the effect of these SNPs on cognitive function in the Singapore population, where information of such associations is sparse. (Fig 1) To the best of our knowledge, the association between these polymorphisms and cognitive function among Singapore multi-ethnic population are unknown. Hence, the aim of this study was to evaluate the associations between pro-inflammatory cytokine gene polymorphisms namely, *IL6-174* (rs1800795 G>C) and *TNF-308* (rs1800629 G>A), and CACI among early-stage breast cancer patients in Singapore. In addition, the differential effect of these SNPs on plasma IL-6 and TNF-α levels, and the associations of plasma IL-6 and TNF- α levels with CACI were also assessed.

## Materials and Methods

### Study design

Patients were recruited at the National Cancer Centre of Singapore (NCCS) and KK Women’s and Children’s Hospital (KKH) in Singapore. This was a prospective, longitudinal, cohort study approved by the SingHealth Centralized Institutional Review Board (IRB), and written informed consent was obtained from all participants.

### Inclusion and exclusion criteria

Eligible patients fulfilled the following criteria: (i) at least 21 years of age diagnosed early-stage breast cancer (stages I to III) by a medical oncologist, (ii) scheduled to receive chemotherapy, either doxorubicin with cyclophosphamide (AC) or docetaxel with cyclophosphamide (TC), (iii) had no prior chemotherapy and/or radiotherapy, (iv) had Eastern Cooperative Oncology Group (ECOG) score of 0 or 1, and (v) were fluent with either English or Chinese.

Patients were excluded if they were: (i) documented with brain metastasis and/or any neuropsychiatric disorders that might result in poor cognitive abilities, (ii) acute illness (e.g. fever), and (iii) physically or mentally incapable of giving written informed consent.

Brain imaging was performed for patients diagnosed with stage III breast cancer, and all study participants were confirmed by their treating medical oncologists for the absence of brain metastasis and leptomeningeal disease.

### Study procedures

Data was collected at 3 independent time points: baseline (T1; before the initiation of chemotherapy), during chemotherapy (T2; 6 weeks after the initiation of treatment), and post-chemotherapy (T3; 12 weeks after the initiation of treatment).

Before the initiation of chemotherapy, clinical characteristics of participants (age, height/weight, race, marital status, education level, and occupation), medical information (cancer diagnosis, cancer stage, comorbidities, ECOG) and medical history (chemotherapy regimen, dose intensity, duration) were obtained from the electronic medical records and through interviews of participants. At each time point of data collection, patients completed two types of neuropsychological assessment tools using Headminder™ (provided by Headminder, Inc. to assess objective cognitive function) and FACT-Cog (to assess self-perceived cognitive function). Furthermore, health-related quality of life (HRQoL), anxiety, and fatigue were also assessed using patient reported outcome questionnaires [9–12]. All cognitive assessments were available in English or Chinese, and were administered by trained bilingual interviewers. For each time point, all assessments took roughly 40 minutes for participants to complete.

### Cytokine analysis and Genotyping

At each time point, a 10-ml blood was collected from the volunteers prior to the receiving of chemotherapy, and stored in an ethylene diamine tetraacetic acid (EDTA) tube. Within 30 to 40 minutes of collection, the blood sample was centrifuged at 1,140 g (2,500 rpm) for 30 min. The plasma and buffy coat were subsequently stored aseptically at −80°C until analysis.

Prior to cytokine analysis, plasma samples were thawed and centrifuged at 10,000g for 10 min at 40°C. The plasma supernatant was used for detection of TNF-α and IL-6 by using the Bio-Plex pro^TM^ Human cytokine 8-plex assay kit (Bio-rad). Samples were analyzed in triplicates and the assay was performed according to the manufacturer’s instructions. The plate was assayed with the Bio-plex 200 multiplex reader and cytokine concentrations were calculated using the Bio-Plex Manager software v6.0 (Bio-rad).

Genomic DNA was isolated from the buffy coat via the QIAamp DNA Blood Mini Kit (Qiagen). The section containing the polymorphisms of *IL6-174* (rs1800795 G>C) and *TNF-308* (rs1800629 G>A) was amplified by polymerase chain reaction (PCR) using specific and optimized primers. Primers utilized for PCR amplification were as follows: 5’-ATGCCAAAGTGCTGAGTCACTA-3’ (forward), 5’-TCGAGGGCAGAATGAGCCTC-3’ (reverse) for *IL6-174* (rs1800795 G>C), and 5’-TCCTGCATCCTGTCTGGAA-3’ (forward), 5’-CAGCGGAAAACTTCCTTGGT-3’ (reverse) for *TNF-308* (rs1800629 G>A). Genotyping was conducted by AITbiotech and they were blinded to all clinical outcomes.

### Statistical Analysis

All statistical analyses were conducted using Stata Version 14 (StataCorp, 2015).

A simple Friedman test was utilized to evaluate the change in plasma cytokine levels over the three time points. Mann–Whitney U-test was used to compare the plasma cytokine levels between the genotype groups for *IL6-174* and *TNF-308* at each time point.

The association of cognitive impairment, as depicted by the global FACT-Cog score (self-perceived cognitive impairment) and the four Headminder™ cognitive domains (objective), with plasma cytokines concentrations during chemotherapy, was evaluated using the generalized estimating equation (GEE) model. For Headminder™, the reliable change index (RCI) was acquired considering the repeated normative mean and standard error of the difference for adjusting the practice effect. Participants were defined as having cognitive impairment in each individual computerized cognitive domain if the RCI was lower than -1.5. A best-fitted model was subsequently created to delineate the association between cognitive impairment and plasma cytokines concentrations after adjusting for documented clinically relevant variables. Clinical confounders that might influence cognitive function were also included a priori into the GEE model as fixed effects; these variables were age, anxiety, fatigue, body mass index (BMI), chemotherapy regimens group (AC versus TC), insomnia, menopausal status, stage of cancer and years of education. Selection of the appropriate correlation structures in the GEE model was conducted using the quasi-likelihood under the independence model criterion, the structure exhibiting the smallest criterion was considered to be the most desirable.

The genetic association between *IL6-174* (rs1800795 G>C) and *TNF-308* (rs1800629 G>A) polymorphisms with CACI was assessed using logistic regression. For FACT-Cog, the minimal clinically important difference (MCID) is defined based on the reduction of ≥10.6 points in the FACT-Cog total score during T2 or T3 compared with T1. Similar to the GEE model, variables that might influence cognitive impairment were also inserted a priori into the logistic regression model. Results were reported as odds ratios (ORs) and 95% confidence intervals (CIs). All statistical tests were two-sided, and p<0.025 was considered statistically significant.

## Results

### Patient Demographics

Patients’ demographics are summarized in Table 1. Their mean age ± SD was 50.3 ± 8.8 years. They were predominantly Chinese (80.8%), and majority (84.0%) received at least high school education and above. Most participants (93.6%) were ambulatory without restrictions on activities, as reflected by an Eastern Cooperative Oncology Group performance status of 0 (fully ambulatory) at baseline assessment. Eighty-five patients (68.0%) received an anthracycline-based chemotherapy regimen.

**Table 1:** Demographics and clinical information of the patients (N = 125).

n (%)		
Age, y, mean ± SD	50.26 ± 8.82	
Ethnicity	Chinese	101 (80.80)
	Malay	13 (10.40)
	Indian	7 (5.60)
	Others^a^	4 (3.20)
Education	Primary school	20 (16.00)
	Secondary school	58 (46.40)
	Pre-university	25 (20.00)
	Graduate/postgraduate	22 (17.60)
Marital status	Single	29 (23.20)
	Married	85 (68.00)
	Divorced	9 (7.20)
	Widowed	2 (1.60)
Working status	Currently working	69 (55.20)
	Currently not working	56 (44.80)
Cancer stage	1	22 (17.60)
	2	66 (52.80)
	3	37 (29.60)
ECOG performance status	0	117 (93.60)
	1	8 (6.40)
Menopausal status	Pre-menopausal	65 (52.00)
	Post-menopausal	60 (48.00)
Chemotherapy regimen	Doxorubicin-containing	85 (68.00)
	Docetaxel-containing	40 (32.00)
Behavior symptoms, mean ± SD		
Baseline fatigue (BFI total score)^b^	1.54 ± 1.76	
Baseline anxiety (BAI total score)^c^	6.94 ± 6.27	
Baseline insomnia score^d^	23.03 ± 27.70	

Abbreviation: ECOG, Ea Eastern Cooperative Oncology Group.

^a^Others includes 1 Burmese and 3 Filipinos.

^b^BFI total score is 10 points.

^c^BAI total score is 63 points.

^d^Insomina subscale total score is 100 points.

### Occurrence of Objective and Self-Perceived Cognitive Impairment

Based on the MCID of FACT-Cog, 36.8% (n = 46) patients experienced self-perceived cognitive impairment. With regards to the Headminder™ cognitive domains, the most commonly affected domains were memory (32.8%, n = 40) and attention (34.2%, n = 39).

### Genotype and Allele Frequencies

All 125 patients were genotyped for the *IL6-174* (rs1800795 G>C) and *TNF-308* (rs1800629 G>A) polymorphisms (Table 2). Genotype frequencies of *IL6-174* and *TNF-308* did not deviate from the Hardy–Weinberg equilibrium, respectively (p = 0.80, p = 0.97). The GG genotype of *IL6-174* and *TNF-308* constituted 97.6% and 83.2% of the observed genotypes, respectively. The C allele of *IL6-174* and the A allele of *TNF-308* accounted for approximately 1.2% and 8.8% of the observed alleles, respectively.

**Table 2:** Genotype and allele frequencies of the *IL6-174* (rs1800795) and *TNF-308* (rs1800629), (N = 125)

Genetic Variant	Genotype Frequency, n (%)			Allele Frequency n (%)	
***IL6–174 G>C (rs1800795)***	**GG**	**GC**	**CC**	**G**	**C**
Chinese	101 (82.8)	0 (0)	0 (0)	202 (81.8)	0 (0)
Malay	12 (9.8)	1 (33.3)	0 (0)	25 (10.1)	1 (33.3)
Indian	5 (4.1)	2 (66.7)	0 (0)	12 (4.9)	2 (66.7)
Others^*^	4 (4.3)	0 (0)	0 (0)	8 (3.2)	0 (0)
Total	122 (97.6)	3 (2.4)	0 (0)	247 (98.8)	3 (1.2)
***TNF-308 G>A (rs1800629)***	**GG**	**GA**	**AA**	**G**	**A**
Chinese	83 (79.8)	17 (85)	1 (100)	183 (80.3)	19 (86.4)
Malay	12 (11.5)	1 (5)	0 (0)	25 (10.9)	1 (4.5)
Indian	5 (4.8)	2 (10)	0 (0)	12 (5.3)	2 (9.1)
Others^*^	4 (3.8)	0 (0)	0 (0)	8 (3.5)	0 (0)
Total	104 (83.2)	20 (16)	1 (0.8)	228 (91.2)	22 (8.8)

^*^ Others includes 1 Burmese and 3 Filipinos.

### Trajectory of Plasma IL-6 and TNF- α Levels

Across the three-time points, there were significant changes in the plasma IL-6 and TNF-α concentrations (p<0.001), with the highest plasma concentrations occurring at the end of chemotherapy (Table 3). Post-hoc analysis showed that the concentration of IL-6 was significantly higher at end of chemotherapy when compared to T2 and T1 (p<0.001). However, post-hoc analysis of plasma TNF-α concentration across time did not achieve statistical significance at any specific time point.

**Table 3:** Plasma IL-6 and TNF-α concentrations across the three time-points, (N = 125).

*Cytokines*	Cytokine concentrations (pg/mL) Median (interquartile range)			p-value	Post hoc (p-value)*		
	T1	T2	T3		T2 vs. T1	T3 vs. T2	T3 vs. T1
**IL-6**	1.08 (0.00–2.84)	0.97 (0.00–3.31)	2.28* (0.00–5.43)	**< 0.001**	0.39	**< 0.001**	**< 0.001**
**TNF-α**	1.26 (0.00–3.66)	1.49 (0.00–3.54)	2.08 (0.56–3.66)	**< 0.001**	0.29	0.02	0.33

*p-value cut-off for post-hoc analysis: 0.0167.

A subgroup analysis was conducted to evaluate the changes in plasma IL-6 and TNF-α concentrations after stratifying for the *IL6-174* (rs1800795 G>C) and *TNF-308* (rs1800629 G>A) genotypes (Table 4). It was observed that there was a significant increase in plasma IL-6 concentration among patients who were carriers of the GG genotype of *IL6-174*, and pair-wise comparisons showed a higher IL-6 concentration at the end of chemotherapy than T2 and T1. After stratifying for the *TNF-308* genotype, significant increase of plasma TNF-α concentration from T1 to T3 was observed in both GG and GA genotype groups, however, post-hoc analysis did not identify any significant differences in TNF-α between any two time points.

**Table 4:** Plasma IL-6 and TNF-α concentrations across the three time-points, after stratifying for the *IL6-174* and *TNF-308* genotypes (N = 125)

*Cytokines*	Cytokine concentrations (pg/mL) Median (interquartile range)			p-value	Post hoc (p-value)*		
***IL6-174* Genotype**	**T1**	**T2**	**T3**		**T2 vs. T1**	**T3 vs. T2**	**T3 vs. T1**
*GG (n = 122)*	1.08 (0.00–2.84)	0.93 (0.00–3.29)	2.28 (0.00–5.43)	**<0.001**	0.47	**0.001**	**<0.001**
*GC (n = 3)*	0.50 (0.25–3.06)	1.09 (0.83–3.18)	2.49 (1.75–5.30)	0.05	0.29	0.11	0.11
*CC (n = 0)*	-	-	-	NA	NA	NA	NA
***TNF-308* Genotype**	**T1**	**T2**	**T3**		**T2 vs. T1**	**T3 vs. T2**	**T3 vs. T1**
*GG (n = 104)*	1.23 (0.00–3.52)	1.39 (0.00–3.65)	2.05 (0.57–3.66)	**<0.001**	0.18	0.32	0.02
*GA (n = 20)*	0.98 (0.00–4.50)	1.70 (0.00–2.63)	2.65 (0.22–3.22)	**<0.001**	0.44	0.57	0.85
*AA (n = 1)*	8.81	58.03	44.62	NA	NA	NA	NA

*p-value cut-off for post-hoc analysis: 0.0167. NA = Not applicable

### Genetic Association of IL6-174 and TNF-308 Polymorphisms with CACI

For both self-perceived and objective cognitive impairment, no associations were established with *IL6-174* and *TNF-308* polymorphisms (S1 and S2 Tables).

### Association of Plasma IL-6 and TNF- α Levels with CACI

Higher plasma IL-6 concentrations were strongly related to severity of self-perceived cognitive impairment (p = 0.001) (Table 5). Both anxiety and insomnia were also strong determinants of self-perceived cognitive impairment; patients with higher levels of anxiety and insomnia reported more cognitive impairment (p<0.001, p = 0.003), respectively.

**Table 5:** Association of IL-6 concentrations with severity of self-perceived cognitive impairment (FACT-Cog) and the cognitive domains of Headminder™.

	FACT-Cog		Headminder™							
	Global score (n = 125)		Attention (n = 114)		Memory (n = 122)		Processing speed (n = 115)		Response speed (n = 122)	
	Coefficient (SE)	p-value	Coefficient (SE)	p-value	Coefficient (SE)	p-value	Coefficient (SE)	p-value	Coefficient (SE)	p-value
Constant	123.696 (11.080)	<0.001	-5.483 (1.916)	0.004	-1.518 (1.900)	0.425	-1.190 (3.624)	0.600	-1.850 (2.624)	0.481
IL-6	-0.036 (0.011)	**0.001**	0.004 (0.003)	0.177	-0.002 (0.004)	0.638	-0.015 (0.026)	0.560	0.002 (0.002)	0.298
Age	0.285 (0.165)	0.085	0.060 (0.028)	**0.032**	0.005 (0.028)	0.853	0.099 (0.056)	0.075	-0.028 (0.036)	0.435
Anxiety	-1.079 (0.131)	**<0.001**	-0.016 (0.028)	0.566	-0.037 (0.032)	0.253	-0.139 (0.084)	0.098	0.024 (0.033)	0.461
Fatigue	-0.381 (0.485)	0.423	-0.029 (0.101)	0.775	0.296 (0.103)	**0.004**	0.336 (0.213)	0.114	0.182 (0.118)	0.123
Body Mass Index	-0.102 (0.238)	0.670	0.104 (0.039)	**0.008**	0.018 (0.038)	0.640	0.011 (0.068)	0.868	0.182 (0.053)	0.883
Chemotherapy group	0.122 (2.563)	0.962	-0.809 (0.460)	0.079	0.039 (0.434)	0.928	-1.907 (0.990)	0.054	0.018 (0.569)	0.975
Insomnia	-0.090 (0.030)	**0.003**	0.019 (0.007)	**0.003**	-0.016 (0.007)	**0.035**	-0.014 (0.015)	0.350	-0.005 (0.008)	0.525
Menopausal status	0.340 (2.871)	0.906	-0.897 (0.501)	0.074	0.013 (0.488)	0.979	-0.433 (0.905)	0.632	0.067 (0.629)	0.916
Years of education	0.387 (0.312)	0.215	0.021 (0.050)	0.670	-0.056 (0.052)	0.280	-0.092 (0.097)	0.344	-0.006 (0.070)	0.930
Breast Cancer stage	0.573 (1.761)	0.745	-0.598 (0.304)	**0.049**	-0.177 (0.294)	0.548	-1.216 (0.596)	**0.041**	0.035 (0.377)	0.925

No associations were established between plasma IL-6 levels and the cognitive domains of Headminder™. However, stage of breast cancer was associated with objective impairment in the domains of attention and processing speed (p = 0.049 and 0.041, respectively). Patients who were older, with higher BMI and insomnia scores had significant association with poorer attention (p = 0.032, 0.008, and 0.003, respectively). Both fatigue and insomnia were also strong determinants of poorer memory (p = 0.004 and 0.035, respectively).

In contrast to IL-6, plasma TNF-α levels were not associated with both self-perceived and objective cognitive impairment.

## Discussion

This study evaluated the genetic associations of *IL6-174* (rs1800795 G>C) and *TNF-308* (rs1800629 G>A) with CACI and cytokine fluctuations within an Asian early-stage breast cancer population. Both *IL6-174* (rs1800795 G>C) and *TNF-308* (rs1800629 G>A) polymorphisms are highly monomorphic in the Singaporean population. In addition, plasma IL-6 and TNF-α concentrations did not differ significantly among the various genotypes, suggesting that both SNPs are not the main factors that drive the over-expression of plasma IL-6 and TNF-α concentrations after initiation of chemotherapy. Although we were unable to establish the genetic associations with cognitive impairment, we have observed that patients manifesting higher levels of IL-6 were associated with greater severity of self-perceived cognitive impairment.

Previous studies have evaluated the downstream effects of *TNF-308* G>A (rs1800629) and *IL6-174* G>C (rs1800795) cytokine gene polymorphisms on the expression of the cytokine levels, as well as the onset of various human illnesses. One study suggested that asthmatic patients who were carriers of the A allele (AA and AG genotypes) of the *TNF-308* G>A (rs1800629) genetic polymorphism had significantly higher serum TNF-α levels, which was also significantly associated with metabolic syndrome [13]. Similar findings were also observed in other inflammatory conditions including depression and inflammatory bowel disease [14, 15]. On the contrary, current evidence to evaluate the impact of these polymorphisms on cognitive function is limited. One recent cross-sectional study has evaluated the associations between the two SNPs and memory complaint among breast cancer patients. It was found that the overall genetic risk index was associated with memory complaint, however, an examination of the individual contribution of each SNP did not identify any significant association with memory complaint [16]. This is in consistent with the current findings where no associations were established between the SNPs and cognitive impairment among chemotherapy receiving breast cancer patients.

In this study, the absence of association between the *TNF-308* (rs1800629 G>A) polymorphism and cognitive decline could be attributed to the low allele frequency of the *TNF-308* G/A polymorphism in our study population, which comprises mainly of Chinese patients. The low frequency of the risk allele was also reported in other genetic association studies that were conducted in the Han Chinese populations [17, 18]. Nevertheless, it must be emphasized that the lack of association between *TNF-308* G/A polymorphism and CACI impairment does not undermine the role of this polymorphism in influencing the expression of TNF-α. In our analysis, we have also observed that plasma TNF-α levels were much elevated (8.81 to 58.03 pg/ml) in one patient who was a homozygous carrier for the risk allele. This finding is similar to another study where the AA genotype frequency was approximately 0.8% (n = 2), and it was observed that TNF-α levels were highly elevated among patients whose genotype was homozygous recessive [19]. Similar to our findings, they were also unable to establish an association between the *TNF-308* G/A polymorphism and disease status. Taken together, these findings suggest that *TNF-308* G/A polymorphism is not the factor influencing circulating TNF-α levels in the majority of our local population, but this could be different in populations with a higher prevalence of the risk allele.

In contrast to the *TNF-308* G/A polymorphism, the major allele of *IL6-174* G/C polymorphism is associated with higher levels of IL-6 [20, 21]. Hence, one would expect to observe higher plasma IL-6 levels among patients who are carriers of the G allele, and the resultant increase in IL-6 levels would be associated with cognitive impairment. Even though the current study did observe an association between plasma IL-6 levels and self-perceived cognitive impairment, we did not observe a dose response change of plasma IL-6 levels for each addition of the minor allele due to the dominance of the GG genotype (98.8%) in the local population. Henceforth, the absence of association between the *IL6-174* G/C polymorphism and cognitive impairment could be attributed largely to the monomorphic nature of the SNP in the local population.

Conversely, dysregulation of cytokines could be attributed to a number of conditions, such as the malignancy, the anticancer treatments, as well as the physical and psychological stressors [22, 23]. In particular, cell death and injury that are caused by chemotherapeutic agents, coupled with both intrinsic and extrinsic stressors, could lead to the peripheral activation of the inflammatory response. IL-6 and TNF-α are primary pro-inflammatory cytokines that could exhibit an acute phase response to tissue injury, infection, or inflammation [24]. The release of peripheral pro-inflammatory cytokines could perturb cytokine levels in the central nervous system, and leads to alterations in neuronal function. In the current study, plasma IL-6 levels were associated with self-perceived cognitive impairment. This finding is consistent with prior studies that have drawn an association between fluctuation in plasma IL-6 levels and self-perceived cognitive disturbances [25–27]. Although TNF-α was also reported to be associated with self-reported memory complaints at baseline and longitudinally among breast cancer patients, the current study failed to establish an association between plasma TNF-α levels and cognitive impairment [28]. The inconsistency in results could be attributed to methodological differences. In a cohort study conducted by Ganz et al, the authors evaluated soluble TNF-receptor II levels at 3 months, 9 months and 15 months after completion of primary treatment, whereas the current study measured plasma TNF-α levels during chemotherapy. It must be emphasized that the biological activity of TNF-α could be limited by its relatively short half-life. Soluble TNF-receptors, on the other hand, have been shown to function as a buffer system to prolong the biological effects of TNF-α. As such, TNF-receptors could be more essential for monitoring inflammatory responses associated with TNF-α.

In line with other studies, cytokine levels (IL-6) were strongly associated with self-perceived cognitive impairment [29, 30]. Dysregulation of pro-inflammatory cytokines are often observed in patients who are experiencing stress-induced symptoms. In our study, we have also identified other stressors such as anxiety and insomnia that are contributing to self-perceived cognitive impairment. Hence, besides measuring how cognitive complaints could subtly impact daily life, self-perceived cognitive impairment is also useful to reflect the degree of emotional and physical symptoms that are experienced among patients [31]. On the other hand, cytokine levels were not associated with impairment in any Headminder^TM^ domains, as objective neuropsychological testing merely captures the cognitive status of a patient at the point of testing [29]. In summary, in epidemiological studies that aim to evaluate the role of pro-inflammatory cytokines on cognitive impairment, it would be prudent to include both subjective and objective measures of cognition as both measures possess different significance.

There are a number of limitations in this study. A number of genetic variations associated with IL-6 and TNF-α were not evaluated in the current study. For example, the *IL6-634* SNP has been shown to be associated with higher circulating serum IL-6 levels [32]. It must be emphasized that the two selected SNPs in this study were based on the functional consequences such as fatigue, depression, and memory complaints that were previously established in the breast cancer population [8]. Higher levels of these cytokines were also strongly associated with cognitive impairment in chemotherapy-breast cancer patients [26, 27]. Hence, we selected these two candidate SNPs in order to establish genuine epidemiological association in our own population. Future studies should evaluate the impact of these other polymorphisms on circulating IL-6 and TNF-α levels, and the resultant association with CACI. Recent studies have also suggested that mitochondrial dysfunctions are correlated with oxidative stress and inflammation in cognitive disorders such as Alzheimer’s disease. [33–37] Future studies should also evaluate how mitochondrial dysfunctions may contribute to CACI in patients with cancer.

## Conclusion

Present findings further contribute to the growing evidence that supports the role of the pro-inflammatory cytokine IL-6 in the occurrence of cognitive impairment post-chemotherapy. However, genetic polymorphism of these cytokines did not play a major role to the cytokine fluctuations as well as cognitive impairment in this cohort. Currently, the lack of understanding of the biological mechanisms underpinning CACI remains the major impediment to the development of effective management strategies. With an increasing evidence to support the cytokine hypothesis, future studies should investigate the role of anti-inflammatory agents in mitigating the cognitive impairment associated with chemotherapy.

## Supporting Information

S1 Table Associations between the *IL6-174* and *TNF-308* gene polymorphisms and the cognitive domains of Headminder™ (DOCX)

S2 Table Associations between the *IL6-174* and *TNF-308* gene polymorphisms and the cognitive domains of FACT-Cog (DOCX)
