# Supplementary material for: Impact of TNF-α (rs1800629) and IL-6 (rs1800795) Polymorphisms on Cognitive Impairment in Asian Breast Cancer Patients
Source: PLoS One. 2016 Oct 4;11(10):e0164204. doi: 10.1371/journal.pone.0164204 (PMC5049844; doi:10.1371/journal.pone.0164204)
Supplement: S1 Table — (DOCX) [file pone.0164204.s001.docx]

|  | *IL-6* (rs1800795) | | | *TNF* (rs1800629) | | |
| --- | --- | --- | --- | --- | --- | --- |
| Domains | Genotype | Adjusted OR (95% CI) | P-value^†^ | Genotype | Adjusted OR (95% CI) | P-value^†^ |
| Attention^*^ | GC | reference |  | GG | reference |  |
|  | GG | - | - | GA | 1.11 (0.29 to 4.34) | 0.88 |
|  |  | - | - | AA | - | - |
| Memory^*^ | GC | reference |  | GG | reference |  |
|  | GG | 1.09 (0.06 to 19.06) | 0.95 | GA | 0.47 (0.13 to 1.65) | 0.24 |
|  |  | - | - | AA | - | - |
| Processing speed^*^ | GC | reference |  | GG | reference |  |
|  | GG | - | - | GA | 2.21 (0.29 to 16.82) | 0.44 |
|  |  | - | - | AA | - | - |
| Response speed^*^ | GC | reference |  | GG | reference |  |
|  | GG | 0.28 (0.01 to 6.38) | 0.43 | GA | 0.87 (0.24 to 3.12) | 0.83 |
|  |  | - | - | AA | - | - |

^*^ORs were calculated using logistic regression under an additive genetic model after adjusting for age, anxiety, fatigue, body mass index, chemotherapy regimen, insomnia, menopausal status, stage of cancer and years of education.

^†^Bolded *p-*values indicate statistical significance, p < 0.025.

CI, confidence interval; OR, odds ratio.
