# Supplementary material for: Impact of TNF-α (rs1800629) and IL-6 (rs1800795) Polymorphisms on Cognitive Impairment in Asian Breast Cancer Patients
Source: PLoS One. 2016 Oct 4;11(10):e0164204. doi: 10.1371/journal.pone.0164204 (PMC5049844; doi:10.1371/journal.pone.0164204)
Supplement: S2 Table — (DOCX) [file pone.0164204.s002.docx]

|  | | | | | | |
| --- | --- | --- | --- | --- | --- | --- |
|  | *IL-6* (rs1800795) | | | *TNF* (rs1800629) | | |
| Domains | Genotype | Adjusted OR (95% CI) | P-value^‡^ | Genotype | Adjusted OR (95% CI) | P-value^‡^ |
| Global score^*^ | GC | Reference |  | GG | Reference |  |
|  | GG | 0.51 (0.03 to 9.21) | 0.65 | GA | 0.46 (0.12 to 1.71) | 0.25 |
|  |  |  |  | AA | - | - |
| Concentration^†^ | GC | Reference |  | GG | Reference |  |
|  | GG | - | - | GA | 0.37 (0.08 to 1.73) | 0.21 |
|  |  |  |  | AA | - | - |
| Functional interference^†^ | GC | Reference |  | GG | Reference |  |
|  | GG | 0.50 (0.02 to 11.60) | 0.66 | GA | 0.30 (0.05 to 1.89) | 0.20 |
|  |  |  |  | AA | - | - |
| Memory^†^ | GC | Reference |  | GG | Reference |  |
|  | GG | - | - | GA | 0.29 (0.05 to 1.57) | 0.15 |
|  |  |  |  | AA | - | - |
| Mental acuity^†^ | GC | Reference |  | GG | Reference |  |
|  | GG | 1.02 (0.06 to 17.57) | 0.99 | GA | 0.48 (0.12 to 1.87) | 0.29 |
|  |  |  |  | AA | - | - |
| Multitasking ability^†^ | GC | Reference |  | GG | Reference |  |
|  | GG | 0.87 (0.05 to 14.54) | 0.92 | GA | 0.87 (0.25 to 3.04) | 0.83 |
|  |  |  |  | AA | - | - |
| Verbal fluency^†^ | GC | Reference |  | GG | Reference |  |
|  | GG | - | - | GA | 0.37 (0.06 to 2.10) | 0.26 |
|  |  |  |  | AA | - | - |

^*^ORs were calculated using logistic regression under an additive genetic model after adjusting for age, anxiety, fatigue, chemotherapy regimen, education level, ethnicity, insomnia, menopausal status, stage of cancer and working status.

^†^ORs were calculated using logistic regression under an additive genetic model after adjusting for age, anxiety, fatigue, chemotherapy regimen, education level, ethnicity, insomnia, stage of cancer and menopausal status

^‡^Bolded *p-*values indicate statistical significance, p < 0.025.

CI, confidence interval; FACT-Cog, Functional Assessment of Cancer Therapy-Cognitive Function; OR, odds ratio.
